# Multi-omics analysis of the mechanism of alfalfa and wheat-induced rumen flatulence in Xizang sheep

**DOI:** 10.1128/spectrum.03268-24

**Published:** 2025-03-07

**Authors:** Jing Wu, Xiaoming Zhang, Khan Ayesha, Shahzad Khuram, Jianzhao Cui, Gaofu Wang, Zhaxi Yangzong, Mingyan Shi, Xunping Jiang, Long Li, Guiqiong Liu, Wangsheng Zhao, Tianzeng Song

**Affiliations:** 1 Institute of Animal Science, Xizang Academy of Agricultural and Animal Husbandry Science, Lhasa, Xizang, China; 2 School of Life Science and Engineering, Southwest University of Science and Technology, Mianyang, Sichuan, China; 3 Interdisciplinary Research Centre in Biomedical Materials (IRCBM), COMSATS University Islamabad (CUI), Lahore, Pakistan; 4 Shigatse Science Popularization Center, Shigatse, Xizang, China; 5 Chongqing Academy of Animal Science, Chongqing, China; 6 College of Life Science, Luoyang Normal University 58292 , Luoyang, Henan, China; 7 College of Animal Science and Technology, Huazhong Agricultural University 47895 , Wuhan, China; University of Arkansas Fayetteville, Fayetteville, Arkansas, USA

**Keywords:** rumen flatulence, rumen microorganisms, Xizang sheep, alfalfa, wheat

## Abstract

Rumen flatulence is a diet-related rumen disease in ruminants. This study induced a rumen flatulence model in Xizang sheep using alfalfa (HRF) and wheatgrass (MRF). The aim was to understand the rumen microbiota diversity in healthy and pathological states, host-microbiota interactions, and the molecular mechanisms of rumen flatulence. Results showed that the pH in the HRF and MRF groups was lower than that in the natural grass group (LRF). SCFA concentrations varied between groups: in HRF, 2-BA and CA increased; in MRF, 4-MVA and 5-MCA rose. Microbial analysis indicated that the alpha- and beta-diversity of HRF and MRF groups were lower than LRF’s, with different microbial compositions. Transcriptome analysis revealed many differentially expressed genes (DEGs). Compared to MRF, HRF had 348 upregulated and 511 downregulated DEGs. Versus LRF, MRF had 201 upregulated and 185 downregulated DEGs, while HRF had 128 upregulated and 238 downregulated DEGs. Spearman’s correlation analysis showed that there was a positive correlation between *Butyrivibrio*, *Quinella,* and specific genes. These findings reveal the potential mechanism of rumen flatulence in Xizang sheep and provide new insights into the prevention and treatment of the disease.

**IMPORTANCE:**

The research used a high-protein diet to induce a model to understand the diversity of rumen microbiota and its interaction with the host, as well as exploring the molecular mechanisms of rumen flatulence.

## INTRODUCTION

Xizang sheep are one of the three original sheep breeds in China and are primarily distributed in the Qinghai-Tibet Plateau and surrounding areas (1). Having lived for generations at high altitudes and in low-oxygen environments, primarily through grazing, Xizang sheep are influenced by various factors that affect their rumen microorganisms. Long-term grazing can lead Xizang sheep to inadvertently consume certain grasses rich in protein and energy, predisposing them to rumen diseases.

Rumen flatulence is typically a fatal digestive system disease that primarily occurs in ruminants fed with leguminous plants or wheat pastures. Leguminous forages contain high concentrations of digestible proteins, which are released into the rumen and lead to the rapid proliferation of rumen microorganisms and fermentation, further resulting in the production of large amounts of gas in the rumen (2, 3). The rapid proliferation of microorganisms, especially the formation of bacterial biofilms, leads to the production of excessive bacterial mucus (4), thereby increasing the viscosity of rumen contents and fermentation gases (5). In addition, legumes also possess foaming properties due to glycosides (saponins), which can further promote flatulence (6). Howarth et al. demonstrated that high-protein forages mainly include alfalfa grass, clover grass, and wheat grass, which can promote the proliferation of certain harmful microbial populations in the rumen, thus affecting rumen health and inducing rumen bloating in ruminants (7).

Alfalfa is widely used among legumes for its high nutritional value and yield. Sheep’s rumen content viscosity increases after consuming fresh alfalfa grass (8). Comparing bloated and non-bloated sheep’s rumen contents, the latter has higher mucus dissolution activity while the former has more and darker foam (9). Even after 24 hours of fasting, there is still some foam in the rumen (10). Alfalfa is an important livestock feed in both developed and developing countries due to its high protein content and high dry matter yield (11). Its main anti-nutritional component is saponins, which limit nutrient absorption in ruminants (6). Lu et al. showed that alfalfa saponins may affect rumen microbial protein synthesis negatively (12). Microorganisms cannot use saponins effectively, indicating an antagonistic relationship between them (6). Previous studies have found that the proportion of plant protein in wheat forage is high and the content of neutral detergent fiber is low (13), and the ruminant itself lacks the ability to degrade soluble protein (14). Intake of high concentrations of protein will cause a certain burden on the rumen. Therefore, they are considered to be a factor in the formation of bloating.

Currently, there are few reports on rumen flatulence. Most studies focus on basic culture methods or qPCR of classical rumen bacteria (15). However, many rumen microorganisms are unculturable or lack targeted genome amplification and may be overlooked as important contributors to rumen foam and flatulence (16). Studies have shown that grazing increases six major bacterial groups associated with flatulence in the rumen contents of cattle in wheat pastures, indicating that foamy flatulence may be related to specific types of bacterial groups (17). Therefore, this study selected alfalfa and wheat forage to induce a rumen flatulence model of Xizang sheep to simulate the ruminants with flatulence under natural conditions and to explore the mechanisms of action of host and microbiome and transcriptome.

## MATERIALS AND METHODS

### Animal selection and experimental design

In this experiment, 24 healthy male Xizang sheep (provided by Longri Breeding Livestock Farm in Hongyuan County, Aba Prefecture, Sichuan Province) aged 7–8 months, with an average body weight of (21.2 ± 2.5) kg were selected and divided into three groups using a completely randomized experimental design. The control group (LRF) grazed freely on natural grass, while the treatment groups consisted of the high-inflation group (HRF), which was fed alfalfa grass, and the moderate-inflation group (MRF), which was fed wheat grass. First, a 7-day pre-test was performed, followed by a 60-day formal test. The formal feeding time was 15 August 2023 (summer). All animals were immunized using standardized procedures. All treatment groups were fed at 8:00 a.m. and 18:00 p.m. every day and free to drink water. On the last day of the experiment, animals were fasting for 8 h. Five Xizang sheep were randomly selected from the control group, and five Xizang sheep with the most obvious bloating were selected from the treatment group for slaughter. Rumen contents and rumen epithelial tissues were collected. Subsequently, the samples were stored in a DNase and RNase-free freezing tube. They were immediately placed in liquid nitrogen, transferred to the laboratory, and kept at −80°C for later analysis.

### Determination of rumen fermentation parameters

The pH value of rumen fluid was measured by a PHBJ-260 portable acidity meter (PHBJ-260, China). First, the acidity meter was corrected with the correction solutions of pH values 4.01, 6.86, and 9.18. In the formal experiment, an appropriate amount of rumen content was taken with a clean beaker, and then the probe of the acidity meter was inserted into the content for direct determination. The concentration of VFA in rumen fluid was determined by gas chromatography (GC-14B gas chromatograph, Japan; capillary column: 30m×0.32mm×0.25 mm; column temperature 110°C, gasification chamber temperature 180°C, and detection chamber temperature 180°C) (18).

### Experimental instruments

The experimental instruments included a QuantStudio 6 Flex fluorescence quantitative PCR instrument (Thermo Fisher Scientific, China), a Qiagen Gel Extraction Kit (Qiagen, Germany), Applied Biosystems Agarose (from Bio-Rad, Hercules, CA, USA), an agarose gel imager (Beijing Wuzhou Technology Co., Ltd.), a 9700 PCR instrument, an agarose electrophoresis system, a Ready gas chromatograph (GC-2010 Plus; Shimadzu, Kyoto, Japan), a 721 spectrophotometer, a centrifuge, a GC-MS instrument, etc.

### DNA extraction

The DNA from the swab samples was extracted using the PureLink Microbiome DNA Purification Kit (Invitrogen, Carlsbad, CA) in accordance with the manufacturer’s instructions. Briefly, the swab samples were treated with Lysis Buffer and Lysis Enhancer. They were then vortexed to enhance cell disruption. After centrifugation, the supernatants were collected, and a Cleanup Buffer was added. The mixture was promptly vortexed again. A second centrifugation was performed to eliminate any sediment. Subsequently, a Binding Buffer was added, and the samples were loaded into spin column-tube assemblies. These assemblies were washed twice with Wash Buffer and then incubated with Elution Buffer. Finally, the resulting mixture was centrifuged to transfer the purified DNA into new sterile centrifuge tubes. The concentration of the DNA was measured using a NanoDrop spectrophotometer (Thermo Fisher Scientific, Inc., Waltham, MA, USA), and the purified DNA samples were stored at −20°C.

### 16S rDNA amplicon sequencing and analysis

The 16S rDNA amplicons from sheep fecal DNA were sequenced by MetWare Biotechnology Co., Ltd. (Wuhan, China) on the Illumina NovaSeq 6000 platform (Illumina, San Diego, CA, USA), following a previously reported protocol (19). Subsequently, the raw data obtained underwent filtering and splicing procedures to generate clean data. Deblur (20) was employed for denoising, resulting in the generation of Amplicon Sequence Variants (ASVs). Species annotation of the ASV sequences was carried out using Mothur (v1.48). Subsequently, taxonomic information and community compositions at multiple levels (phylum, class, order, family, genus, and species) were analyzed. To assess species richness and evenness within the samples, as well as to identify common and unique ASV across different samples, alpha-diversity metrics were calculated. These metrics included the Shannon index, Simpson index, Chao1 index, ACE index, observed ASVs, Goods coverage, and PD whole_tree. For investigating beta-diversity between groups, Principal Coordinates Analysis (PCoA), Principal Component Analysis (PCA), Non-Metric Multi-Dimensional Scaling (NMDS), and the Unweighted Pair-group Method with Arithmetic Means (UPGMA) were utilized. These analyses were based on the Weighted UniFrac distance of ASV abundances. Differences in microbial composition and community structure between the two groups were statistically analyzed using the t-test and Wilcoxon test. In addition, Tax4Fun2 (v1.1.5) was adopted to predict the functions of microbes. The Spearman correlation coefficient was applied to evaluate the relationships between microbes and differentially expressed genes (DEGs), as well as inter-microbial relationships.

### RNA extraction, cDNA synthesis, and real-time quantitative PCR

Total RNA was extracted from rumen samples with RNAiso Plus reagent (Takara Biotechnology Co., Ltd., Dalian, China) following the standard extraction protocol. Subsequently, the isolated mRNA was reverse-transcribed into cDNA using the PrimeScript RT kit (Takara) equipped with gDNA Eraser. For real-time quantitative PCR (qPCR), TB Green Premix Ex Taq II (Tli RNaseH Plus) was utilized, and the reactions were carried out in a CFX96 real-time PCR detection system (Bio-Rad, Hercules, CA, USA). The relative gene expressions were determined by applying the 2^−ΔΔCt^ method.

### RNA sequencing and analysis

RNA sequencing was performed by MetWare Biotechnology Co., Ltd. (Wuhan, China) using the Illumina NovaSeq 6000 platform (Illumina, San Diego, California, USA). The raw data were filtered to obtain clean data, which was then compared with the reference genome of Xizang sheep using Hisat2 (21), https://ftp.ncbi.nlm.nih.gov/genomes/all/GCF/000/298/735/GCF_000298735.2_Oar_v4.0/. DEGs among the three groups were analyzed using FeatureCounts and DESeq (22). The false discovery rate (FDR) was calculated using the Benjamini-Hochberg method to adjust the *P*-values. DEGs were identified based on |log2Fold Change| ≥ 1 and FDR < 0.05. Data analyses, such as heat map generation, volcano plot construction, Gene Ontology (GO), and Kyoto Encyclopedia of Genes and Genomes (KEGG) pathways analyses, were performed using the Metware Cloud platform. (https://cloud.metware.cn).

### Statistical analyses

The data were expressed as mean ± standard error (SEM). They were then analyzed using GraphPad Prism 8. To determine the differences between the two groups, an unpaired Student’s t-test was applied. In this study, a significance level of *P* < 0.05 was considered indicative of a significant difference, with * denoting *P* < 0.05, ** denoting *P* < 0.01, and *** denoting *P* < 0.001.

## RESULTS

### Comparison of rumen fluid fermentation parameters among three groups of Xizang sheep

**Fig 1:**
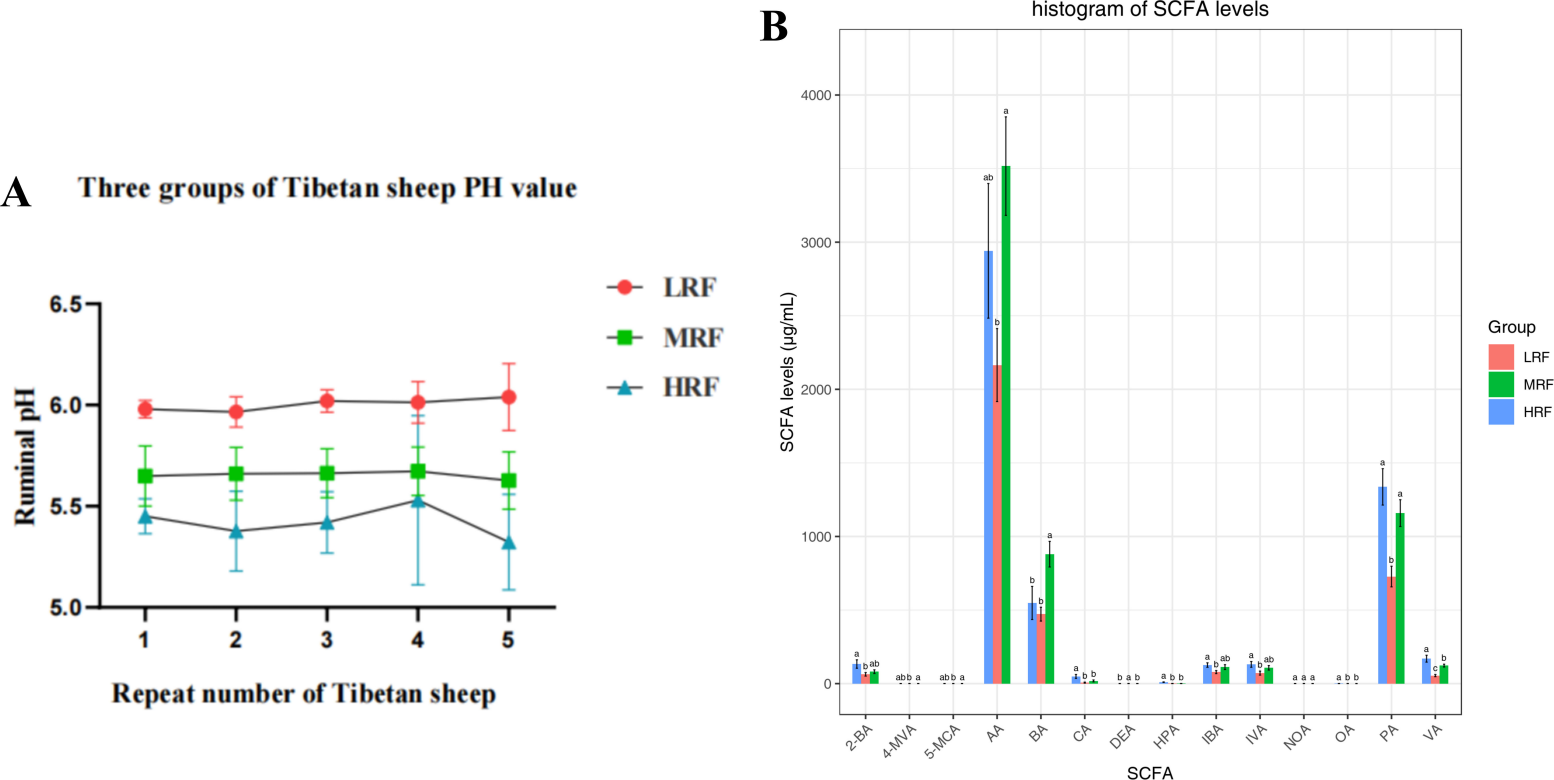
Comparison of rumen fermentation parameters of three groups of Xizang sheep. (**A**) Rumen PH. (**B**) short-chain fatty acids.

Changes in rumen pH values are shown in Fig. 1A, and rumen fermentation parameters are shown in Fig. 1B. The results showed that compared with the LRF group, the rumen pH values in the MRF and HRF groups were significantly reduced, with *P*-values of 0.0063 and 0.0003 respectively. In addition, compared with the LRF group, the concentrations of isocaproic acid (4-MVA), isoheptanoic acid (5-MCA), acetic acid (AA), butyric acid (BA), propionic acid (PA), and valeric acid (VA) were significantly increased in the MRF group. In the HRF group, the concentrations of 2-methylbutyric acid (2-BA), caproic acid (CA), heptanoic acid (HPA), isobutyric acid (IBA), isovaleric acid (IVA), octanoic acid (OA), PA, and VA increased significantly. Only the concentration of decanoic acid (DEA) was significantly higher in the LRF group than in the other two groups, while the concentration of nonanoic acid (NOA) did not differ significantly among the three groups.

### Changes in α- and β-diversity of rumen microflora in Xizang sheep

**Fig 2:**
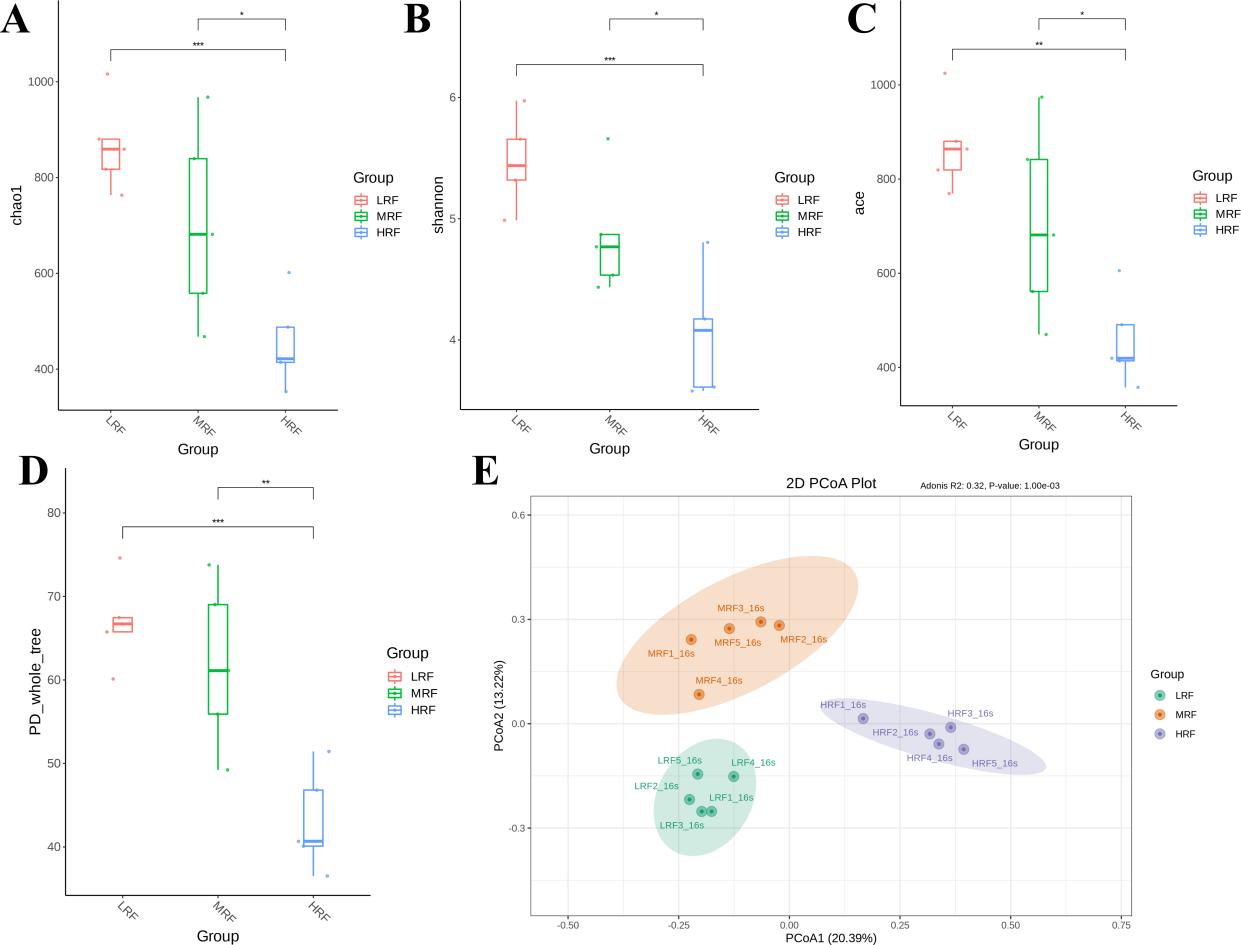
Comparison of α-diversity changes and β-diversity of rumen microbial communities among three groups of Xizang sheep. (**A**) Chao1. (**B**) Shannon. (**C**) ACE. (**D**) PD-whole-tree. (**E**) PCoA. ⁎ denotes significant, ⁎⁎ denotes highly significant, ⁎⁎⁎ denotes extremely significant, LRF represents non-bloated Xizang sheep, MRF represents moderately bloated Xizang sheep, and HRF represents severely bloated Xizang sheep.

The richness and diversity of the microbial community were mainly measured by the Chao1 index, Shannon index, PD_whole_tree index, and ACE index (Fig. 2A through D). The α- and β-diversity of rumen microbiota varied with diet. The Chao1 index, Shannon index, PD_whole_tree index, and ACE index of rumen flora in the Xizang sheep of the LRF group were significantly higher than those in the HRF group (*P* < 0.01), but there was no significant difference between the LRF group and the MRF group. The Chao1 index, Shannon index, PD_whole_tree index, and ACE index of rumen microflora in the MRF group were significantly higher than those in the HRF group (*P* < 0.05). These results indicate that the diversity of rumen flora in Xizang sheep is greatly affected by diet. As shown in Fig. 2E, principal coordinates analysis (PCoA) revealed significant differences in the β-diversity of rumen microbiota among the three groups of Xizang sheep. The results demonstrated that the abundance and diversity of rumen microflora in the LRF group and MRF group were significantly higher than those in the HRF group. This indicates that the sensitivity of rumen flatulence induced by alfalfa is higher, and such a high flatulence rumen environment is not conducive to the survival of microorganisms.

### Changes in rumen microflora composition in Xizang sheep

**Fig 3:**
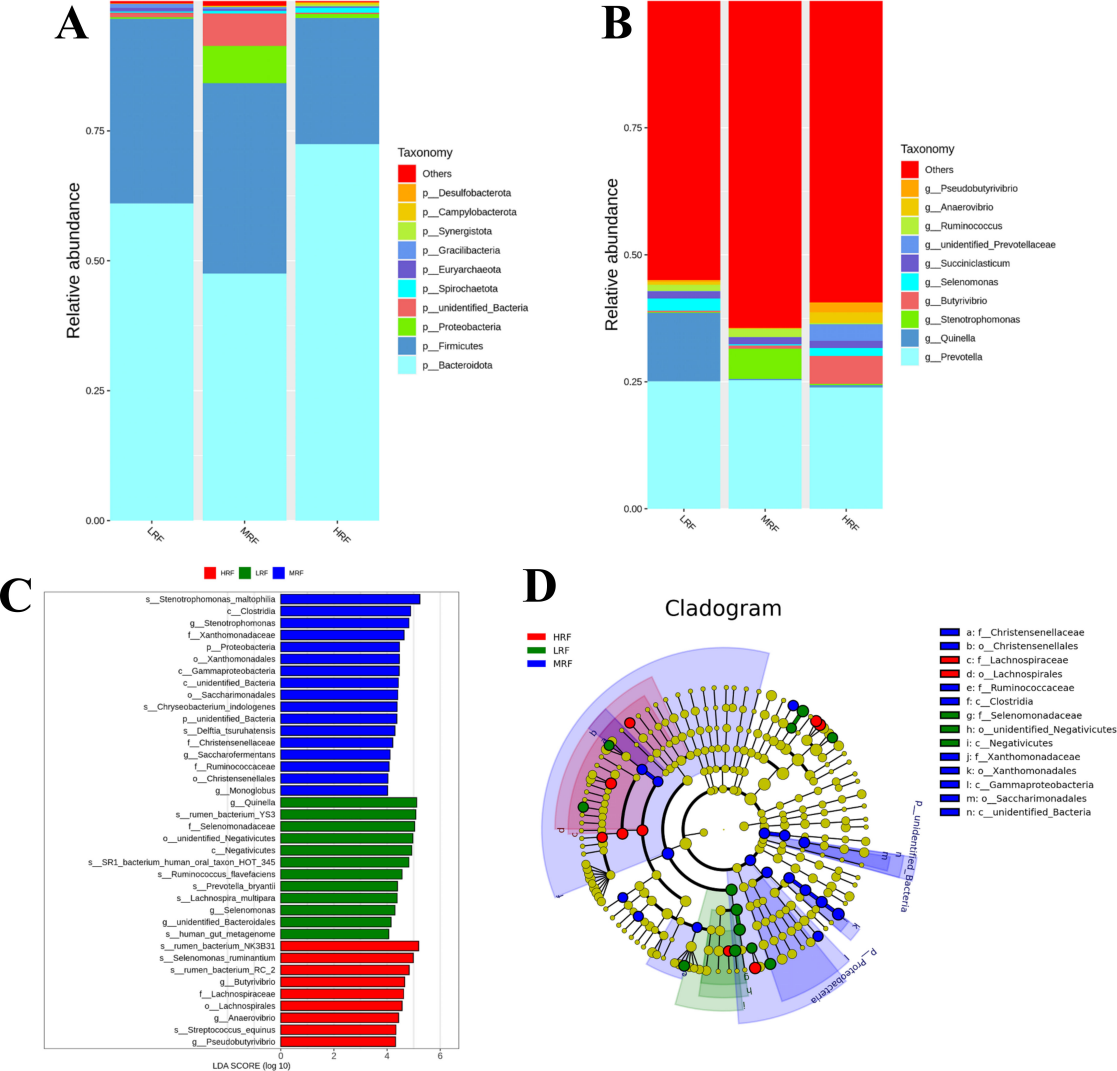
Variations in rumen microflora composition of Xizang sheep. (**A**) Composition of the microbial community at the phylum level and non-bloating groups. (**B**) Composition of the microbial community at the genus level and non-bloating groups. (**C**) Phylogenetic distribution from phylum to genus level of the microbial community, indicated by the branching diagram generated from LEfSe analysis. (**D**) LDA score histogram was used to identify the differentially abundant bacterial genera between Xizang sheep and non-bloating groups.

At the microbial phylum-level classification, the relative abundances of the top 10 rumen microflora in Xizang sheep from the LRF, MRF, and HRF groups were analyzed, as shown in Fig. 3A. Among these, Bacteroidota was the dominant phylum, accounting for 61.0%, 47.5%, and 72.4% in the three groups, respectively. Firmicutes was the second most abundant, making up 35.5%, 36.6%, and 24.2% in the three groups, respectively. Notably, the HRF group had the highest abundance of Bacteroidota and the lowest abundance of Firmicutes among the groups; these are the two largest phyla of rumen microorganisms in Xizang sheep. At the microbial genus level, as shown in Fig. 3B, *Prevotella* was common across all groups, with relative abundances of 25.1% in LRF, 25.3% in MRF, and 23.9% in HRF. In addition, *Quinella* accounted for 13.4% of the dominant bacteria in the LRF group but only 0.3% and 0.4% in the MRF and HRF groups, respectively. *Stenotrophomonas* was more dominant in the MRF group at 5.9% compared to 0.2% and 0.3% in the LRF and HRF groups, respectively. By contrast, *Butyrivibrio* was predominant in the HRF group at 5.4%, with lower abundances of 0.2% and 0.6% in LRF and MRF, respectively.

LEfSe (LDA Effect Size), an analytical tool for biomarker discovery and interpretation across subgroups, was employed to identify differentially enriched bacterial groups among the LRF, MRF, and HRF groups (Fig. 3C), using an LDA score threshold of 4 (Fig. 3D). This study identified 38 genera as key differentiators. Characteristic microflora in the MRF group included *Stenotrophomonas_maltophilia*, *Clostridia*, *Stenotrophomonas*, *Xanthomonadaceae*, *Proteobacteria*, *Xanthomonadales*, *Gammaproteobacteria*, *unidentified_Bacteria*, *Saccharimonadales*, *Chryseobacterium_indologenes*, *unidentified_Bacteria*, *Delftia_tsuruhatensis*, *Christensenellaceae*, *Saccharofermentans*, *Ruminococcaceae*, *Christensenellales*, and *Monoglobus*. The LRF group was characterized by *Quinella*, *rumen_bacterium_YS3*, *Selenomonadaceae*, *unidentified_Negativicutes*, *Negativicutes*, *SR1_bacterium_human_oral_taxon_HOT_345*, *Ruminococcus_flavefaciens*, *Prevotella_bryantii*, *Lachnospira_multipara*, *Selenomonas*, *unidentified_Bacteroidales*, and *human_gut_metagenome*. In the HRF group, the characteristic microflora included *rumen_bacterium_NK3B31*, *Selenomonas_ruminantium*, *rumen_bacterium_RC_2*, *Butyrivibrio*, *Lachnospiraceae*, *Lachnospirales*, *Anaerovibrio*, *Streptococcus_equinus*, and *Pseudobutyrivibrio*. A hierarchical classification diagram from the phylum to the species level showed significant phylogenetic differences among the three groups. These findings suggest that differences in dietary structure may lead to variations in the rumen microflora composition among the groups, with significant enrichment of certain rumen microflora potentially contributing to rumen flatulence in Xizang sheep.

### The construction and sequencing of the transcriptome library

**Fig 4:**
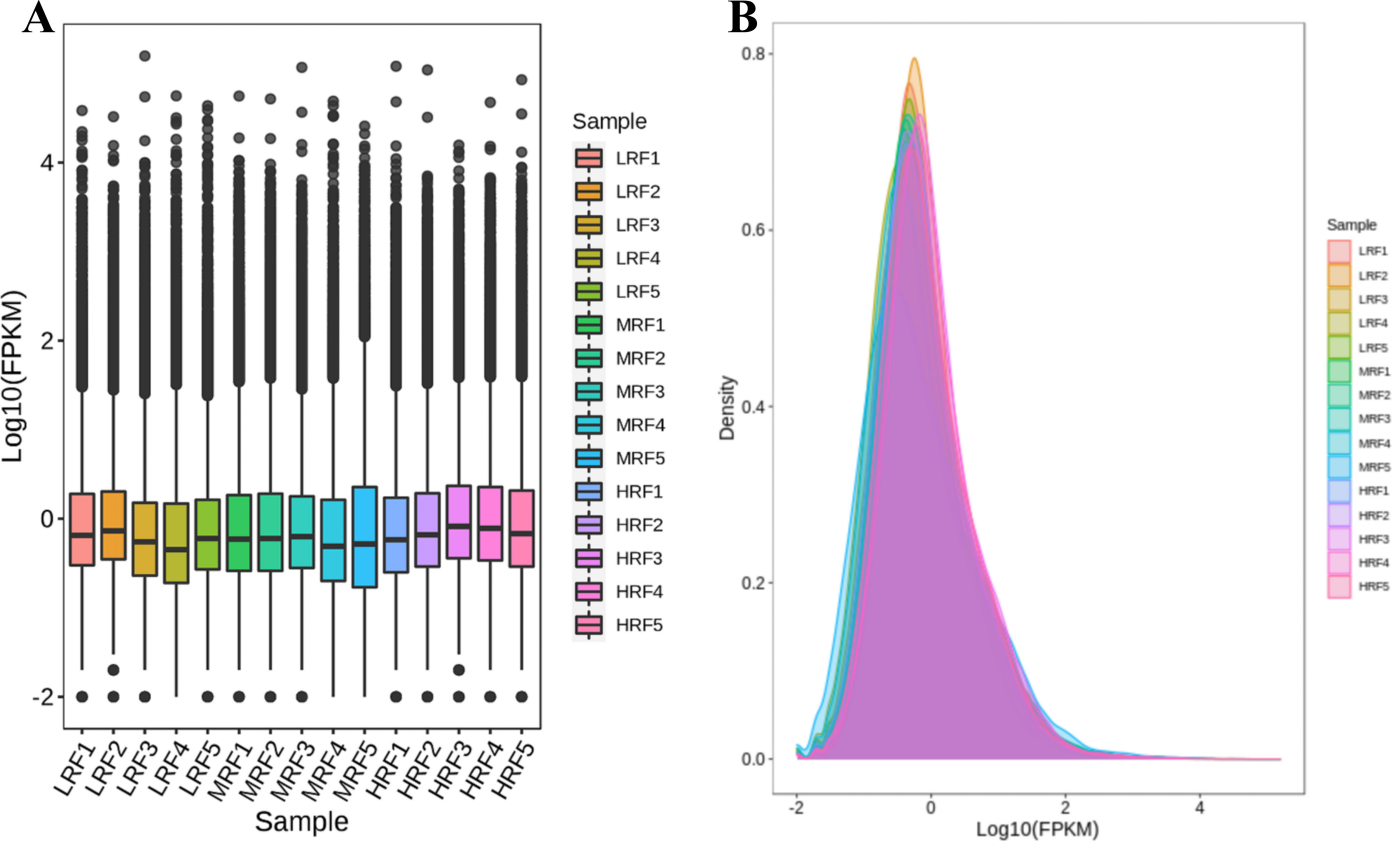
DEGs among the three groups of Xizang sheep. (**A**) Overall gene expression levels and trends of expression in each group. (**B**) Clustering of DEGs among the groups.

Box plots and density plots were utilized to display the variability and abundance of gene expression levels in individual samples (Fig. 4A and B) for the RNA-sequencing analysis of 15 rumen-epithelium specimens, namely LRF1, LRF2, LRF3, LRF4, LRF5, MRF1, MRF2, MRF3, MRF4, MRF5, HRF1, HRF2, HRF3, HRF4, and HRF5.

### Comparative analysis of DEGs

#### DEGs identified in the rumen epithelium of LRF and HRF

**Fig 5:**
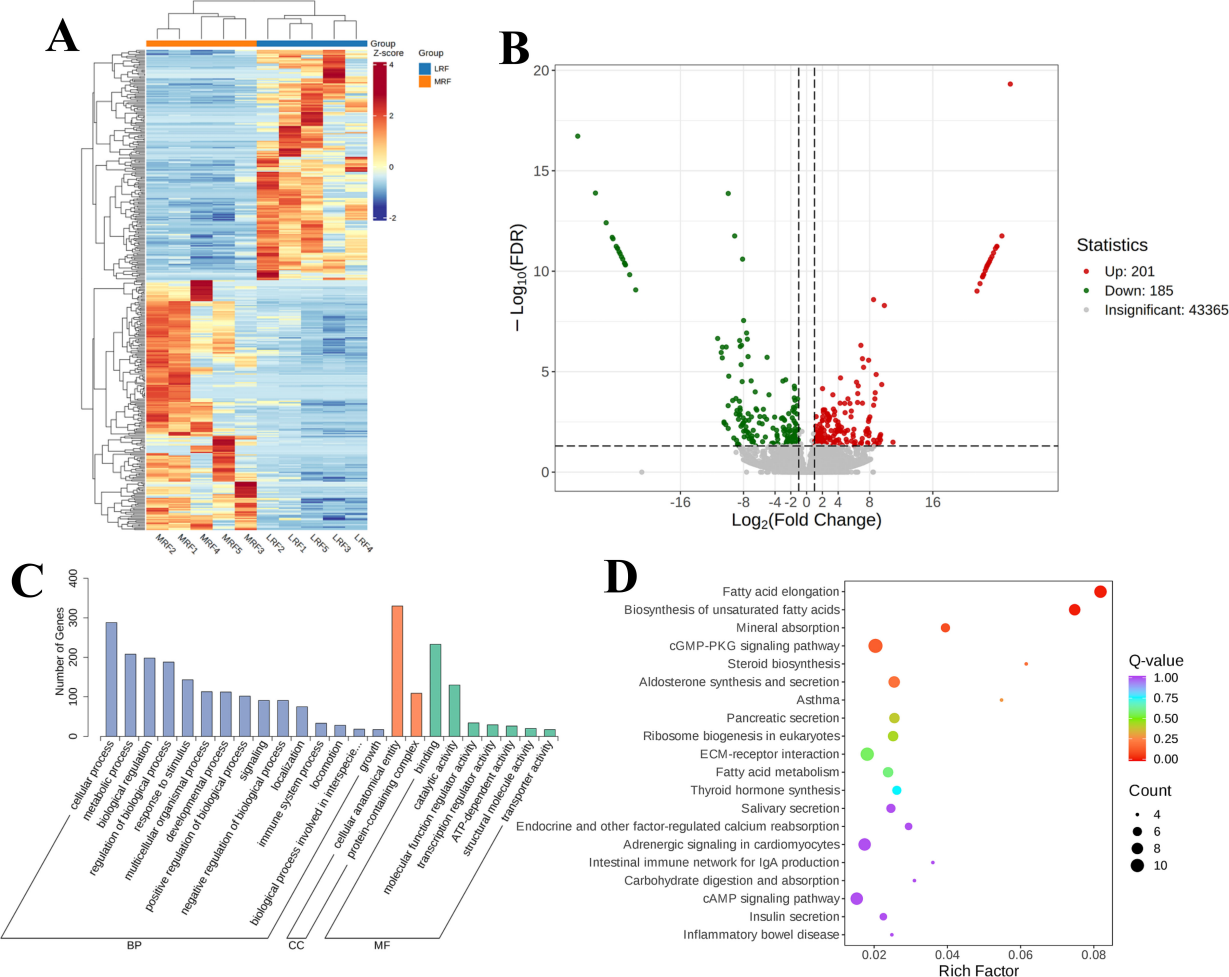
DEGs in the rumen epithelium of Xizang sheep between LRF and HRF groups. (**A**) Clustering of DEGs. (**B**) Volcano plot of DEGs. (**C**) GO classification diagram. (**D**) KEGG enrichment plot of all the DEGs.

Further clustering analysis of the DEGs between the LRF and HRF groups revealed distinct clustering patterns of highly and lowly expressed genes in the rumen tissue samples. Heatmap analysis identified 859 shared DEGs between the LRF and HRF rumen-epithelium samples (Fig. 5A and B). Specifically, compared with the LRF group, the HRF group exhibited 348 upregulated and 511 down-regulated DEGs. GO enrichment analysis was performed to predict the functional properties of these DEGs (Fig. 5C). The results showed that the DEGs observed in the rumen epithelium of the LRF and HRF groups were enriched in biological processes (BP) such as cellular process, metabolic process, response to stimulus, multicellular organismal process, and regulation of biological process, with significant differences between the two groups. In terms of cellular components (CC), the DEGs were involved in cellular anatomical entities and protein-containing complexes. Regarding molecular functions (MF), the DEGs were primarily concentrated in binding and catalytic activity. To further explore the biological functions of the DEGs, a KEGG pathways analysis was conducted to assess pathway differences. As shown in Fig. 5D, the significantly enriched pathways included carbon metabolism (2.23%) and biosynthesis of amino acids (1.45%), with the metabolism pathway showing the highest gene enrichment (9.95%).

#### DEGs identified in the rumen epithelium between LRF and MRF

**Fig 6:**
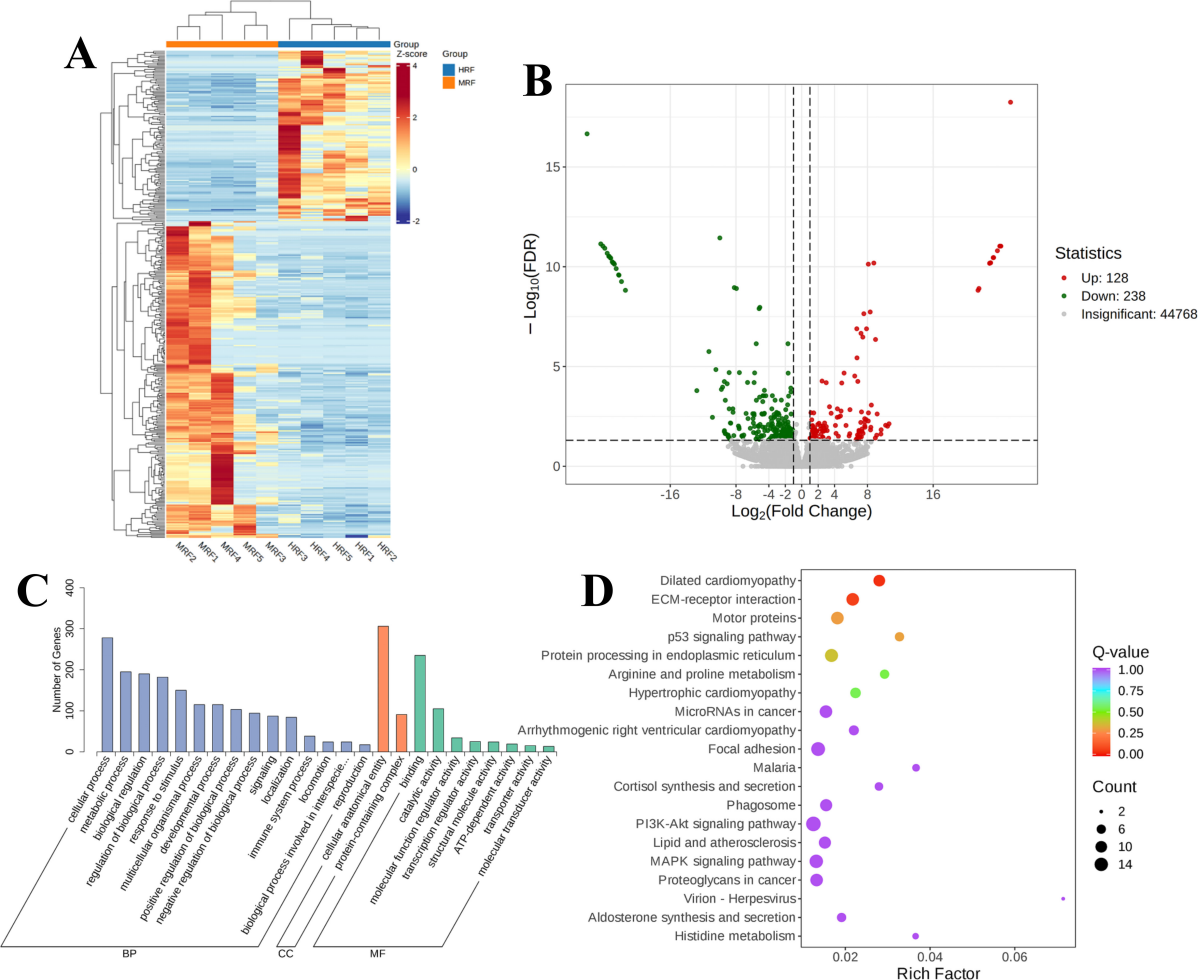
DEGs in the rumen epithelium of Xizang sheep between LRF and MRF Groups. (**A**) Clustering of DEGs. (**B**) Volcano plot. (**C**) GO classification diagram. (**D**) KEGG enrichment plot of all the DEGs.

Further clustering analysis of the DEGs between the LRF and MRF groups (Fig. 6A) revealed significant clustering of highly and lowly expressed genes in the rumen tissue samples from each group. A heatmap diagram showed that there were 386 common DEGs between the LRF and MRF rumen-epithelium samples (Fig. 6B). Specifically, compared with the LRF group, the MRF group exhibited 201 upregulated and 185 downregulated DEGs. GO functional enrichment analysis was conducted to identify the functions of the DEGs in the rumen epithelium of Xizang sheep in the LRF and MRF groups, providing insights into their functional roles. As shown in Fig. 6C, the analysis focused on BP, CC, and MF. In BP, the DEGs were primarily involved in cellular processes, metabolic processes, responses to stimuli, multicellular organismal processes, and the regulation of biological processes. In CC, the DEGs were involved in cellular anatomical entities and protein-containing complexes. In MF, the DEGs were primarily concentrated in binding and catalytic activities. To determine the biological functions of the DEGs, a KEGG pathway enrichment analysis was performed to determine whether specific pathways were significantly enriched. As shown in Fig. 6D, the significantly enriched pathways included the cGMP-PKG signaling pathway (6.32%), fatty acid elongation (5.17%), biosynthesis of unsaturated fatty acids (4.6%), aldosterone synthesis and secretion (4.6%), ECM-receptor interaction (5.75%), adrenergic signaling in cardiomyocytes (5.17%), and the cAMP signaling pathway (5.17%). The pathways with the highest gene enrichment were the metabolism and organismal systems pathways.

#### DEGs identified in the rumen epithelium between HRF and MRF

**Fig 7:**
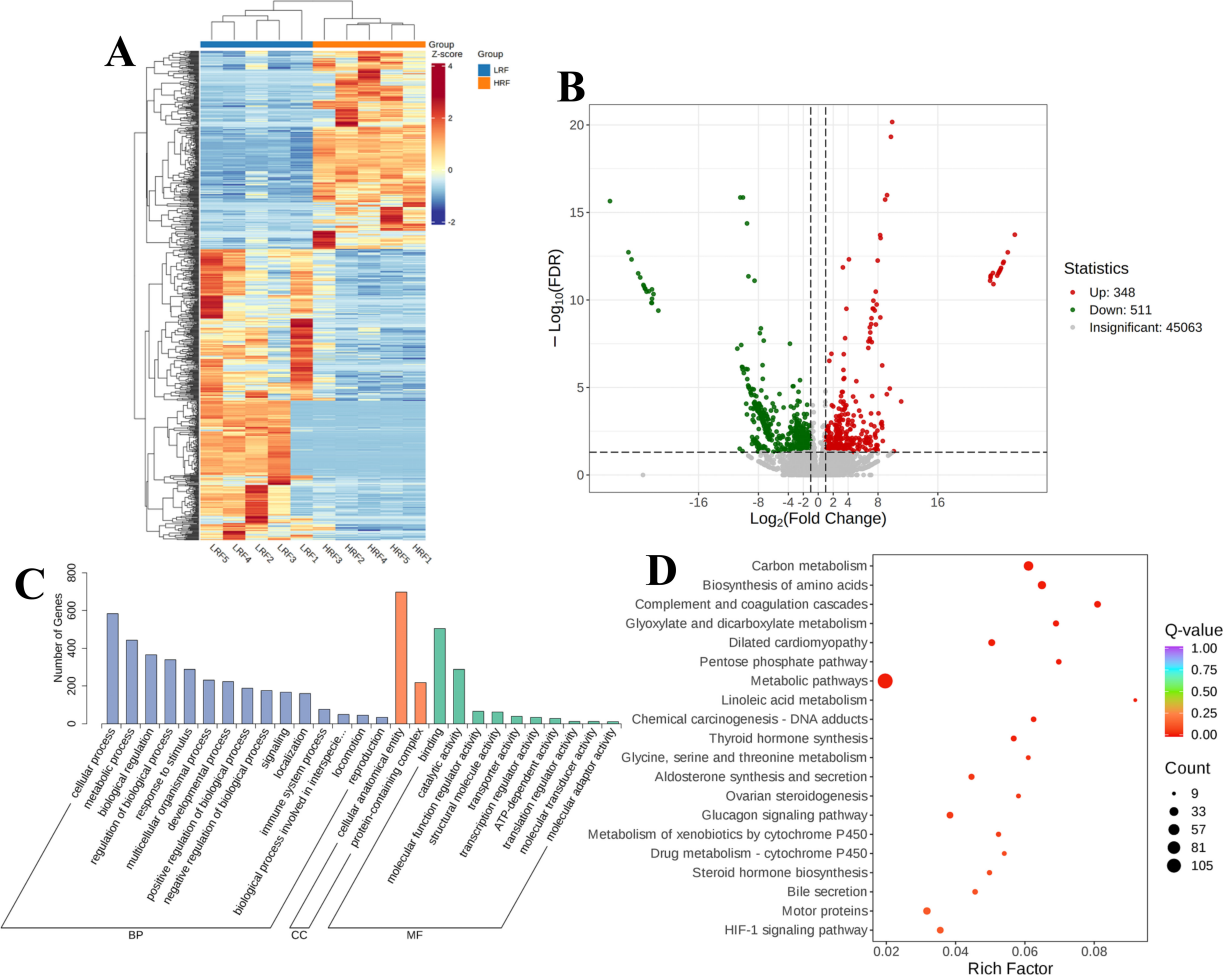
DEGs in the rumen epithelium of Xizang sheep between LRF and MRF Groups. (**A**) Clustering of DEGs. (**B**) Volcano plot. (**C**) GO classification diagram. (**D**) KEGG enrichment plot of all the DEGs.

Further clustering analysis of the DEGs between the HRF and MRF groups (Fig. 7A) revealed distinct clustering of highly and low-expressed genes in the rumen tissue samples from each group. Heatmap analysis identified 366 common DEGs between the HRF and MRF rumen-epithelium samples (Fig. 7B). Specifically, compared with the HRF group, the MRF group exhibited 128 upregulated and 238 downregulated DEGs. To understand the functions of the DEGs, GO term functional enrichment analysis was performed using the DEGs from the HRF and MRF groups in the rumen epithelium. As shown in Fig. 7C, the analysis focused on BP, CC, and MF. Within the BP category, the DEGs were mainly involved in cellular processes, metabolic processes, responses to stimuli, multicellular organismal processes, regulation of biological processes, and biological regulation. In the CC category, the DEGs were involved in cellular anatomical entities and protein-containing complexes. In the MF category, the DEGs were primarily concentrated in binding and catalytic activities. To determine the biological functions of the DEGs, a KEGG pathways enrichment analysis was conducted to identify any significantly enriched pathways. As indicated by Fig. 7D, the significantly enriched pathways included dilated cardiomyopathy (5.52%), ECM-receptor interaction (6.63%), motor proteins (6.63%), protein processing in the endoplasmic reticulum (7.18%), microRNAs in cancer (6.63%), adrenergic signaling in cardiomyocytes (5.17%), focal adhesion (8.29%), PI3K-Akt signaling pathway (9.39%), MAPK signaling pathway (7.73%), and proteoglycans in cancer (6.63%). The pathway with the highest gene enrichment was the Human Diseases pathway.

### Correlation analysis between rumen microbiota and differentially expressed genes

**Fig 8:**
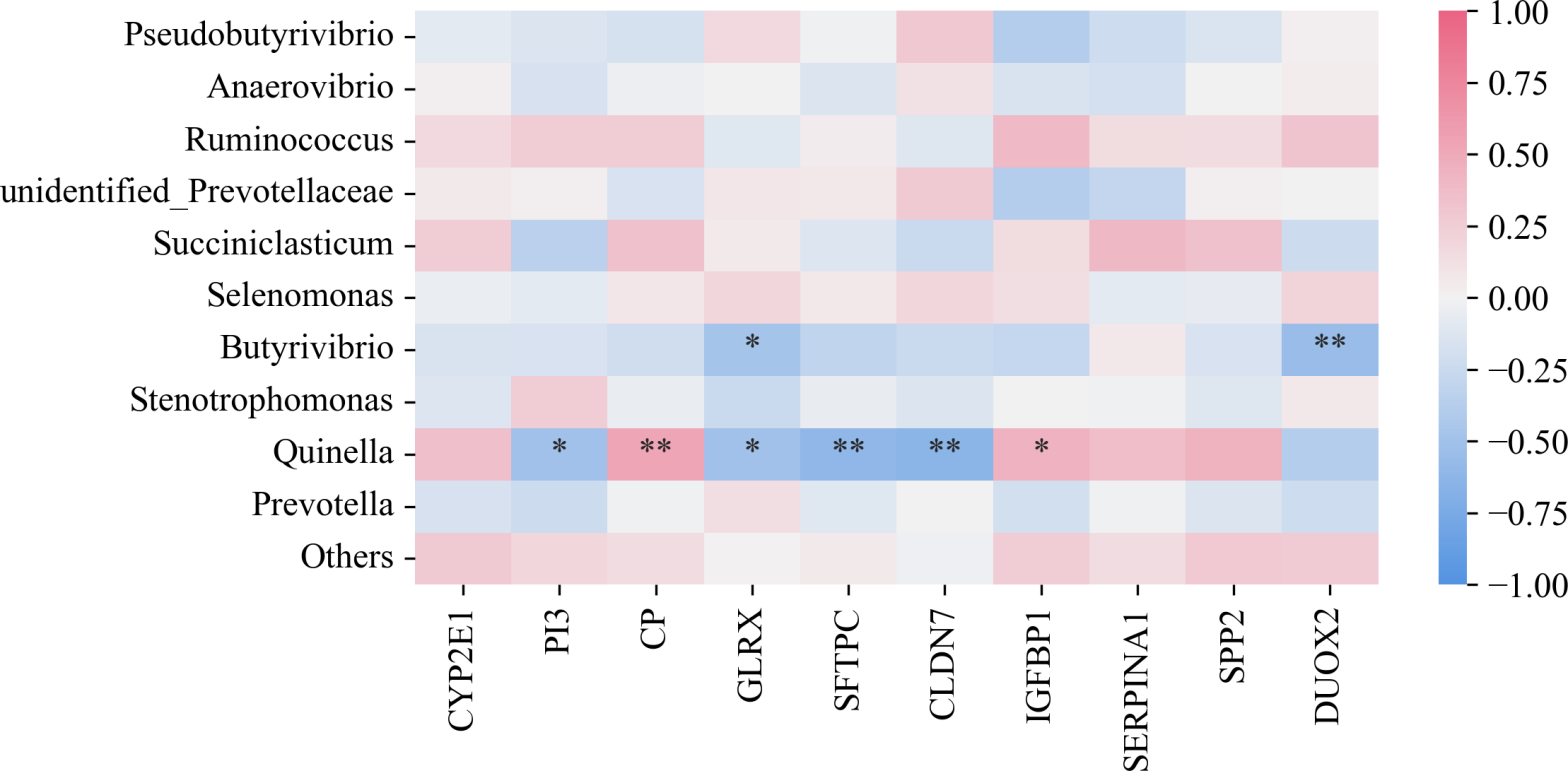
Correlation analysis between rumen microbiota and differentially expressed genes. ⁎denotes significant, ⁎⁎denotes highly significant.

The Spearman correlation heatmap was utilized to analyze the relationship between rumen microbes and DEGs in the rumen, aiming to explore the effects of different diets on the host. As shown in Fig. 8, a significant negative correlation was observed between the *Butyrivibrio microbiota* and the *GLRX* and *DUOX2* genes. *Quinella* was significantly negatively correlated with *PI3*, *GLRX*, *SFTPC*, and *CLDN7* genes, while it was positively correlated with *CP* and *IGFBPI*. Therefore, it can be inferred that the species most affected included *Butyrivibrio* and *Quinella*, which were identified as the dominant genera in the HRF and LRF groups, respectively.

### RT-qPCR validati

**Fig 9:**
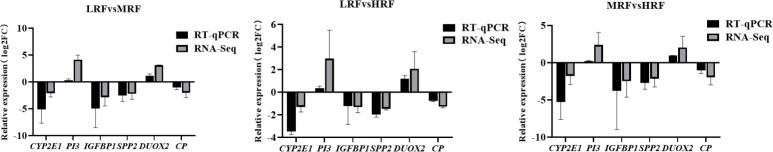
Verification of differentially expressed genes.

To confirm the reproducibility and reliability of gene-expression levels in the RNA-sequencing analysis, six randomly selected DEGs, namely *CYP2E1*, *PI3*, *IGFBP1*, *SPP2*, *DUOX2*, and *CP*, were validated using qRT-PCR. The results were consistent with those of the RNA-sequencing findings (Fig. 9).

## DISCUSSION

In recent years, the development of omics technologies has enabled researchers to explore the host-microorganism relationships more deeply. Many studies have shown that the composition and function of the gastrointestinal microbiota affect health, disease, metabolism, immunity, and neurobehavior (23). Meanwhile, certain genes are associated with disease and immunity, highlighting their importance in maintaining health (24). This study used rumen transcriptomics and microbiology techniques to investigate the host-microbiota interactions in Xizang sheep rumen and the impact of rumen bloat on them, aiming to explore the regulatory mechanisms of bloat disorders.

Wheat and alfalfa are major forage grasses on the Qinghai-Tibet Plateau. They are drought-resistant, cold-resistant, adaptable, high-yielding, and nutrient-rich, suitable for high-altitude harsh environments (25, 26). These forages are important livestock feed, especially in winter (27, 28). When wheat hay level decreases, protein content increases while feed digestibility declines significantly (29). Ruminants may not fully absorb protein, and undigested protein can abnormally ferment in the gastrointestinal tract, producing harmful by-products that damage gut health and increase disease risk. High-protein diets, like barley feeding, can lead to liver damage in cattle (30) and are associated with bloat and indigestion in ruminants. Previous experiments showed that low-fiber diet-fed animals had bloating or digestive disorders (31). In this study, we induced rumen bloat in Xizang sheep using wheat and alfalfa and analyzed the causes by comparing rumen microbiota diversity and rumen epithelium differentially expressed genes among groups.

The rumen, a crucial digestive organ in ruminants, is easily affected by diet. Dietary changes can alter ruminal pH and volatile fatty acids (VFAs) (32). In this study, the pH of bloat-induced Xizang sheep fed wheat and alfalfa was significantly lower than that of non-bloat sheep, consistent with ruminal acidosis results (33, 34). The accumulation of VFAs in the rumen increases osmotic pressure, potentially damaging ruminal epithelial cells and affecting metabolism and health (35). Dietary protein and fiber levels mainly determine rumen-disrupting components (36). Diet affects rumen fermentation patterns. Volatile fatty acids, such as acetate, propionate, and butyrate, are major metabolic products of ruminal microbes’ anaerobic fermentation, providing 70%–80% of ruminants’ energy (37). In addition, the concentration of VFAs is an important indicator of ruminal fermentation efficiency. There are significant differences in dietary composition between grazing and housed systems (38), pasture-based diets typically have higher crude protein (CP) and soluble carbohydrate levels, with lower starch content (39). In our experiment, the concentrations of acetate, propionate, and butyrate in bloat-induced Xizang sheep were significantly higher than those in non-bloat sheep, indicating different feeds’ significant impact on ruminal fermentation. Wheat’s fiber and structural carbohydrates may increase these VFA levels (40), and alfalfa’s high protein and easily fermented non-structural carbohydrates also contribute to VFA production (41).

The rumen has a large number of microorganisms forming a complex ecosystem (42, 43). Alpha-diversity can be used to assess the diversity of bacteria in the rumen of ruminants, with the Shannon index reflecting the evenness and diversity of rumen bacteria (44). Generally, a higher Shannon index indicates greater bacterial diversity within the sample. A larger PD_Whole_tree index and Chao1 index signify higher bacterial abundance in the sample (43). In this study, the Shannon index, PD_Whole_tree index, and Chao1 index showed highly significant differences among the three groups, with the MRF group and HRF group significantly lower than the LRF group. PCoA also revealed significant beta-diversity differences in rumen microbial communities. Previous studies on goats with rumen bloat induced by high-concentrate diets had similar results, indicating rumen bloat’s significant impact on Xizang sheep rumen microbial community diversity. The composition and structure of rumen bacteria are vital for maintaining homeostasis, enhancing nutrient digestion, and supporting health (45). The ratio of Firmicutes to Bacteroidetes is an important indicator of microbial impact on host energy needs (46). In this study, Bacteroidetes and Firmicutes were dominant phyla in the three groups, helping the host adapt to high-altitude habitats. However, the MRF group had a significantly lower abundance of Bacteroidetes and a higher abundance of Firmicutes compared to the other two groups, potentially due to limited wheat nutrient absorption in Xizang sheep rumen. The MRF group also had dominant Proteobacteria and unidentified Bacteria, likely due to wheat forage substances promoting their growth.

*Prevotella* is generally regarded as a dominant genus in the rumen microbiome (47). In this study, the genus *Prevotella* was identified as the dominant genus across all three groups, while *Quinella* was the dominant genus in the LRF group, Stenotrophomonas in the MRF group, and *Butyrivibrio* in the HRF group. *Prevotella* plays a central role in carbohydrate and hydrogen metabolism, and a high abundance of *Prevotella* in ruminants is associated with a healthy microbiome (48). The capacity of *Prevotella* to ferment carbohydrates is also reflected in the productivity of cattle. It has been reported that, in a study involving a group of lactating Holstein cows, animals with higher protein and fat content in their milk exhibited higher concentrations of acetate, butyrate, and propionate in their rumen fluid (49). Furthermore, methane production was positively correlated with VFA concentrations (primarily acetate and butyrate), while rumen pH significantly decreased, which aligns with the findings of this study.

In this study, the genus *Prevotella* was identified as the dominant genus across all groups, with *Quinella* emerging as the dominant genus in the LRF group. The abundance of methanogenic archaea is proportional to the occurrence of rumen bloat. *Quinella*, a hallmark rumen bacterium first reported in 1913, has been shown to increase in number in the rumen of sheep, correlating with lower methane (CH₄) emissions (50). The fact that *Quinella* is the dominant genus in the LRF group, which is less sensitive to bloat, suggests that *Quinella* may play a role in alleviating the occurrence of bloat. However, how this genus contributes to the lower methane emissions in these sheep remains to be investigated. Previous research has indicated that *Stenotrophomonas* is associated with inflammation and tends to increase in the rumen of cows suffering from subacute ruminal acidosis (51). Furthermore, administering *Stenotrophomonas* to lactating mice can induce mastitis. Wheat-based diets have also been linked to health issues in cows due to their high dry matter content, leading to increased levels of *Stenotrophomonas* in their rumen, which positively correlates with acetate, propionate, and total volatile fatty acid (TVFA) levels. Thus, it can be inferred that the high abundance of *Stenotrophomonas* in the MRF group of Xizang sheep subjected to wheat feeding was a result of inflammation induced by the diet, contributing to rumen disorders. Members of the genus *Butyrivibrio* are significant components of the rumen microbiota (52). Early characterization of *Butyrivibrio* has shown that some strains possess the ability to degrade cellulose, while most can metabolize xylan and pectin substrates. It has been reported that ruminal *Butyrivibrio* can utilize various soluble and some insoluble substrates, fermenting carbohydrates into butyrate, formate, lactate, and acetate (53). In addition, *Butyrivibrio* exhibits metabolic versatility and can utilize various insoluble substrates, although it is not classified as a cellulose-degrading bacterium. Studies have indicated that while *Butyrivibrio* hungatei can use oligosaccharides and monosaccharides as growth substrates, it cannot hydrolyze proteins or degrade fibers (54). Therefore, it is hypothesized that the high protein content in alfalfa stimulates substantial *Butyrivibrio* production in the rumen of the HRF group, leading to extensive fermentation of protein and gas production, thereby promoting the occurrence of bloat in Xizang sheep. Overall, the analysis of rumen microbiota in this study indicates that different dietary regimens affect the microbial composition in the rumen of Xizang sheep. These specific microbes can contribute to the development of rumen disorders such as bloat. The identification of dominant genera within each group illustrates their potential role in the occurrence of rumen bloat in Xizang sheep.

Rumen bloat involves a series of encoded genes, and to investigate the regulatory gene changes among the LRF, MRF, and HRF groups, an RNA-seq analysis was conducted. The results revealed a total of 1,417 DEGs across the three groups. Specifically, between the LRF and HRF groups, there were 859 shared DEGs, with 511 downregulated and 348 upregulated; between the LRF and MRF groups, there were 386 shared DEGs, with 185 downregulated and 201 upregulated; and between the HRF and MRF groups, there were 366 DEGs, with 238 downregulated and 128 upregulated. The functional annotation of these DEGs indicated their involvement in cellular processes, metabolic processes, responses to stimuli, multicellular organism processes, biological regulation, cellular component organization, protein complexes, binding, and catalytic activity regulation. By integrating the rumen microbiome with host gene expression patterns, we found correlations between certain rumen microbes and host genes. Research suggests that gene expression may play a mediating role between microbial communities and host functionality. For instance, *CYP2E1* is involved in disease occurrence and immune-mediated mechanisms, with downregulation of *CYP2E1* observed in nearly all immune-mediated diseases, making it a significant factor in disease development (55). Consequently, the downregulation of *CYP2E1* may impair animal health. *CYP2E1* is a critical enzyme in the metabolic activation of various toxic substances, including nitrosamines, benzene, vinyl chloride, and halogenated solvents like trichloroethylene (56). Cows exhibiting signs of ketosis show abnormal expression of *CYP2E1* compared to healthy cows. The oxidation of multiple substrates mediated by *CYP2E1* can release substantial reactive oxygen species, leading to lipid peroxidation and cytotoxicity (57). Therefore, *CYP2E1* serves as a significant marker involved in disease development. The *PI3* signaling pathway is implicated in gastrointestinal regulation. Studies found that gas in the gastrointestinal tract may result from the activation of the PI3K/AKT signaling pathway, with the production of phosphatidylinositol 3-kinase being triggered during such conditions (58). Abnormal activation of the PI3K signaling pathway has been observed across various diseases (59). In this study, the upregulation of *PI3* expression was related to the PI3K/AKT pathway, suggesting that bloat in ruminants may lead to elevated *PI3* expression. When there is an excess of growth hormone or metabolic disorders, levels of *IGFBP1* decrease, potentially resulting in severe physiological phenomena such as abdominal spasms, gastrointestinal bloat, and diarrhea (60). In this study, *IGFBP1* showed a downregulated trend, indicating a potential relationship between gastrointestinal bloat and altered *IGFBP1* levels. Previous research found that the expression of *IGFBP5* did not correlate with the growth and proliferation of rumen papillae in Holstein calves, but *IGFBP5* in rumen papillae indicated subacute acidosis, damaging the gastric epithelial cells in Holsteins (61). Differential expression of *IGFBP3* and *IGFBP6* in non-lactating Holstein cows was reduced due to grain-induced subacute acidosis, resulting in increased concentrations of myocardial lactate and LPS, which harmed gastric epithelial cells (62). Secreted phosphoprotein 2 (*SPP2*) is primarily produced in the liver and transported to other tissues, where it has been identified as a significant bone matrix protein involved in the regulation of bone metabolism (63). *SPP2* may interact with insulin-like growth factor binding protein 1 (*IGFBP1*), potentially inhibiting the progression of liver fibrosis and cirrhosis (64). Previous studies have also reported a consistent and significant decrease in *SPP2* expression in tumors, with reduced *SPP2* levels in hepatocellular carcinoma (HCC) being associated with advanced clinical and pathological features, indicating a potential tumor-suppressive role for *SPP2* in HCC (65). Research has demonstrated that *SPP2* can inhibit tumor growth and induce tumor apoptosis by eliminating the pro-tumor functions of bone morphogenetic protein 2 (*BMP-2*) signaling (66). These findings suggest that *SPP2* and related genes may play a role in disease development, leading to adverse effects in Xizang sheep that are particularly sensitive to bloat.

Through the analysis of the rumen microbiome and host gene expression patterns, we identified multiple associations between differentially expressed rumen epithelial genes in LRF, MRF, and HRF groups of Xizang sheep and the rumen bacteria. Evidence for the relationship between host gene expression and the composition of the rumen microbiome suggests that gene expression may mediate interactions between microbial communities and host functions (67). In this study, a significant negative correlation was found between the genus *Butyrivibrio* and the *GLRX* and *DUOX2* genes. In addition, *Quinella* was negatively correlated with genes *PI3*, *GLRX*, *SFTPC,* and *CLDN7*. It was significantly positively correlated with *CP* and *IGFBP1* genes. Previous research has indicated that reduced *GLRX* expression exacerbates liver fibrosis, with observed decreases in *GLRX* and increases in PSSG in fibrotic mice and human liver samples (68). Furthermore, *GLRX1* deficiency has been shown to make male mice sensitive to high-fat diets (69), with *GLRX1* deficiency worsening liver damage and oxidative stress induced by high-fat diets (70). An increase in *DUOX2* expression affects intestinal immune homeostasis in mice, and mutations in *DUOX2* can lead to hypothyroidism (71). Notably, *Butyrivibrio* is an important degrader and user of lignocellulosic plant materials (72). These results suggest that the sensitivity to bloat in Xizang sheep may be influenced by the interplay of various microbes and differentially expressed host genes.

### Conclusions

In this study, the multi-omics method was used to comprehensively analyze the related changes of rumen flatulence induced by different feeds in Xizang sheep. The results showed that the rumen pH value of Xizang sheep fed with alfalfa and wheat grass decreased, the concentration of volatile fatty acids changed, the ɑ- and β-diversity of rumen microorganisms decreased, and the composition of phylum and genus levels was significantly different. There were a large number of differentially expressed genes in rumen epithelial tissue, and *Butyrivibribrio* and *Quinella* were significantly correlated with specific genes. This reveals that the potential mechanism of rumen flatulence is that specific feed changes the rumen environment, affects the microbial community, and then causes flatulence through the interaction between microorganisms and host gene expression. Subsequent studies may further explore the mechanism through gene editing or microbial intervention, develop prevention and treatment strategies, expand to different ruminants and breeding environments, and combine more multi-omics techniques to comprehensively analyze the mechanism of rumen flatulence.

## Data Availability

All data used in this study are available upon request from the corresponding or first author. In addition, the dataset created specifically for this study has been deposited in the NCBI Sequence Read Archive, which can be found under the BioProject identifiers PRJNA1178088 and PRJNA1180951.
